# The function of nicotinamide phosphoribosyl transferase (NAMPT) and its role in diseases

**DOI:** 10.3389/fmolb.2024.1480617

**Published:** 2024-10-24

**Authors:** Aihong Peng, Junqin Li, Jianxiao Xing, Yuanjun Yao, Xuping Niu, Kaiming Zhang

**Affiliations:** Shanxi Key Laboratory of Stem Cells for Immunological Dermatosis, State Key Breeding Laboratory of Stem Cells for Immunological Dermatosis, Institute of Dermatology, Taiyuan Center Hospital, Taiyuan, China

**Keywords:** NAMPT, NAD metabolism, biological function, SIRTs, diseases

## Abstract

Nicotinamide phosphoribosyl transferase (NAMPT) is a rate-limiting enzyme in the mammalian nicotinamide adenine dinucleotide (NAD) salvage pathway, and plays a vital role in the regulation of cell metabolic activity, reprogramming, aging and apoptosis. NAMPT synthesizes nicotinamide mononucleotide (NMN) through enzymatic action, which is a key protein involved in host defense mechanism and plays an important role in metabolic homeostasis and cell survival. NAMPT is involved in NAD metabolism and maintains intracellular NAD levels. Sirtuins (SIRTs) are a family of nicotinamide adenine dinucleotide (NAD)-dependent histone deacetylases (HDACs), the members are capable of sensing cellular NAD+ levels. NAMPT-NAD and SIRT constitute a powerful anti-stress defense system. In this paper, the structure, biological function and correlation with diseases of NAMPT are introduced, aiming to provide new ideas for the targeted therapy of related diseases.

## 1 Introduction

In 1957, Preiss and Handler the first reported that the identification of NAMPT as an enzyme involved in the biosynthesis of NAD (Garten et al., 2015; Wang and Miao, 2015; Preiss and Handler, 1957). In 1994, the NAMPT coding gene was screened from the cDNA genebank of human peripheral blood lymphocytes for the first time (Samal et al., 1994), and was named as the pre-B-cell colony enhancing factor (PBEF) (Sun et al., 2013). In 2005, it was discovered that NAMPT is highly expressed in visceral adipose tissue, with the NAMPT level of plasma was significantly correlated with the prognosis of obese patients. Consequently, NAMPT has been considered as an adipokine and renamed visfatin (Chang et al., 2011). Although NAMPT, PBEF, and visfatin have been used in the literature, NAMPT is the official name for the protein and gene, approved by the HUGO Gene Nomenclature Committee and the Mouse Genomic Nomenclature Committee (Fukuhara et al., 2005).

NAMPT is widely expressed in human marrow, liver, muscle, and various other organs and tissues. It is also expressed in immune cells, cardiomyocytes, fibroblasts, and neurons, among other cells (Yang et al., 2006). This widespread expression underscores the critical role of NAMPT in both physiological and pathological states (Friebe et al., 2011). NAMPT exists in two distinct forms: extracellular NAMPT (eNAMPT) and intracellular NAMPT (iNAMPT). iNAMPT, a pleiotropic protein, is predominantly localized in the cytoplasm, nucleus, and mitochondria, especially in neurons of the hippocampus and cerebral cortex (Garten et al., 2015). iNAMPT expression is elevated in brown adipose tissue (BAT), liver and kidney; moderate in white adipose tissue (WAT), lung, spleen, testes and skeletal muscle; and undetectable in the brain and pancreas. In the rate-limiting process of NAD, iNAMPT can be used as a key enzyme to catalyze biosynthesis pathway and participate in various biological processes such as energy metabolism, antioxidant reaction, cell proliferation and apoptosis (Chen X. et al., 2015; Liu et al., 2021). eNAMPT performs its role as a growth factor, enzyme and cytokine. NAMPT is an active protein in the extracellular space that promotes the formation of pre-B cell clones and facilitates the maturation of B cells, which is originally called PBEF. Current research indicates that eNAMPT is essential for maintaining tissue homeostasis, enhancing NAD levels, SIRT1 activity, and neural activation in the hypothalamus. It is also a pivotal regulator of inflammatory networks, promoting the release of inflammatory cytokines (Yoshida et al., 2019; Quijada et al., 2021). eNAMPT, believed to be derived from post-translational modification of iNAMPT, primarily released into the plasma from adipose tissue, where it catalyzes the synthesis of NMN. The biological activities of NAMPT have been tested both *in vitro* and *in vivo* (Audrito et al., 2020). The biological functions of NAMPT as a regulator of NAD have been extensively studied *in vitro* (Yang et al., 2006). By regulating the biosynthetic activity of NAD, NAMPT mediates the activity of NAD-dependent enzymes such as acetylase (Garten et al., 2009; Koltai et al., 2010; Pavlová et al., 2015; Chen et al., 2016; Wang and Miao, 2019), poly (ADP ribose) polymerase (Henning et al., 2018), and CD38 (a transmembrane enzyme) (Lee and Aarhus, 1991), thereby influencing cell metabolism, mitochondrial biogenesis, and the adaptive responses to inflammation and oxidative stress (Chen et al., 2016; Galli et al., 2013; Garten et al., 2015). The interplay between NAMPT and SIRT signaling constitutes a robust defense mechanism against various stressors (Wang and Miao, 2015). SIRTs, a family of NAD-dependent histone deacetylases, the activation of which delays the onset of neurodegenerative diseases, have garnered significant attention in the neurological disorders. Previous studies have demonstrated that NAMPT delays aging by enhancing resistance to oxidative stress (Wang et al., 2016).

## 2 The crystal structure of nicotinamide phosphoribosyltransferase (NAMPT)

**FIGURE 1:**
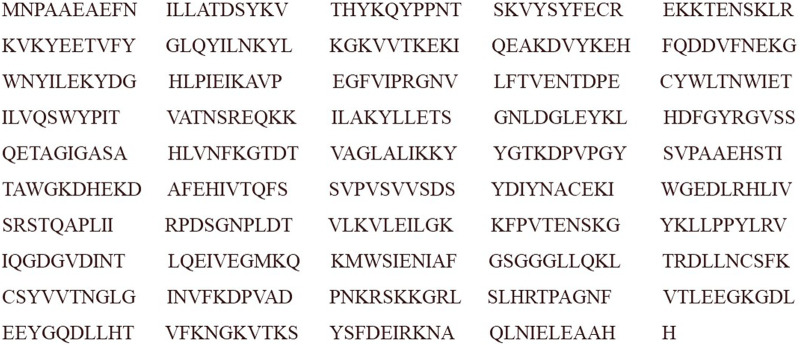
Primary structure of NAMPT. Amino acid sequence of *Homo sapiens*’s NAMPT.

The gene that encodes NAMPT locates on human chromosome seven between 7q22.1 and 7q31.33 with a total length of 34.7 kilobases (kb), and contains 11 exons and 10 introns with a total nucleotide sequence length of 2,357 base pairs (bp) (Wen et al., 2024; Sun et al., 2013). The NAMPT protein is composed of 491 amino acids and has a molecular weight of 52 kDa (kDa) (Figure 1) (Wen et al., 2024). The protein’s structure includes 19 β-chains and 13 α-helices, which are arranged into two distinct domains (Murphy and Bloom, 2006). The NAMPT structure is similar to the nicotinate phosphoribosyltransferase (NAPRTase) and quinolinate phosphoribosyl transferase (QAPRTase) of the hyperthermophilic archaea (Wang et al., 2006). The X-ray crystal structure shows that NAMPT is a homodimeric protein that belongs to a dimer class of type II phosphoribosyltransferase, and the crystal structure of NAMPT in complex with various ligands have been elucidated. These structures typically contain a NAMPT homodimer (Murphy and Bloom, 2006) with two analogous active sites at the dimer interface, where two NMN molecules bind (Murphy and Bloom, 2006). NAMPT inhibitors typically occupy the NAM-binding active site, as well as a typical tunnel-like cavity extending from the NAM-binding site. Notably, many NAMPT inhibitors are unique in that they rely on the cellular efficacy of nitrogenous heterocyclic moieties. When the NAMPT inhibitor binds to the NAMPT protein, the heterocycle components extend into the NAM-binding site and simulate the covalent interaction of the natural substrate with 5-phosphoribosyl-1-pyrophosphate (PRPP) (Khan et al., 2006).

## 3 Biological functions of nicotinamide phosphoribosyltransferase (NAMPT)

In 1957, Preiss and Handler reported that NAMPT can catalyze the synthesis of NMN (Preiss and Handler, 1957). As is well-known, NAMPT participates in the NAD+ metabolism and maintains the levels of intracellular NAD. By regulating the biosynthetic activity of NAD, NAMPT influences the activity of NAD-dependent enzymes, including poly ADP-ribose polymerase (PARP), CD38, and SIRTs. The NAMPT-NAD and SIRTs constitute a powerful anti-stress defense system (Garten et al., 2015). Therefore, NAMPT is implicated in the regulation of various cellular processes, including cell metabolism, mitosis, inflammation, and oxidation stress (Garten et al., 2015). NAMPT can regulate the circadian rhythm of metabolism by mediating SIRT1’s circadian regulators (clock circadian regulator (CLOCK) and brain and muscle arnt-like 1 (BMAL1)) (Galli et al., 2013). The regulatory effect of NAMPT on SIRT has been widely concerned (Ramsey et al., 2009).

**FIGURE 2:**
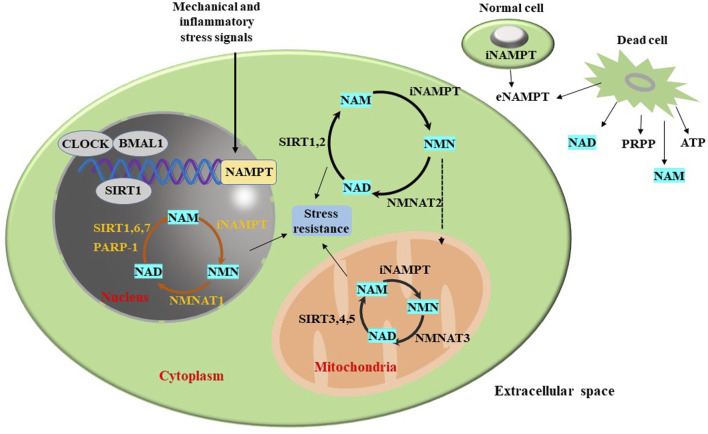
Biological functions of NAMPT.

In addition to the intracellular functions, the extracellular functions of NAMPT have garnered attention. The expression of NAMPT is induced by pathogen-derived lipopolysaccharide (LPS) and host-derived inflammatory cytokines, such as tumor necrosis factor-α (TNF-α), interleukin-1β (IL-1β) and interleukin-6 (IL-6), modulating inflammatory responses (Busso et al., 2008; Audrito et al., 2020; Wang et al., 2021). NAMPT influences the immune response and inhibits apoptosis of immune cells such as neutrophils and macrophages (Travelli et al., 2018). Although the extracellular functional mechanisms of NAMPT have not been definitively elucidated (Carbone et al., 2017), its potential as a therapeutic target has been underscored due to its important physiological functions (Figure 2).

## 4 Nicotinamide adenine dinucleotide (NAD+) metabolism

**FIGURE 3:**
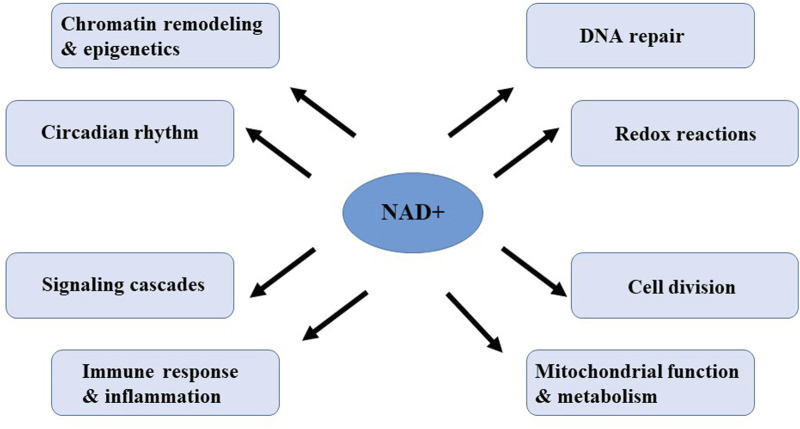
Cellular processes regulated by or dependent on NAD+.

Nicotinamide adenine dinucleotide (NAD+) is a pivotal metabolite and coenzyme in a variety of metabolic pathways and cellular processes, and present in every known form of life (Mori et al., 2014). NAD+ serves as a crucial cofactor for non-redox NAD+ dependent enzymes, including deacetylase, CD38 and poly (ADP-ribose) polymerase (Garavaglia et al., 2012; Grozio et al., 2013; Wilk et al., 2020). NAD+ can directly and indirectly influence numerous key cellular functions, including DNA repair, chromatin remodeling and epigenetics, cell division, immune response and inflammation, mitochondrial function and circadian rhythms, which are critical for maintaining tissue, metabolic homeostasis and healthy aging (Figure 3) (Rajman et al., 2018). Notably, in a variety of model organisms, including rodents and humans, aging is accompanied by a gradual decline in tissues and cellular NAD+ levels (McReynolds et al., 2020). The decline in NAD+ levels is causally linked to many age-related diseases, including cognitive decline, cancer, metabolic disorders, sarcopenia, and frailty. These aging-related diseases can be slowed down or even reversed by restoring NAD+ levels. Therefore, targeting NAD+ metabolism has emerged as a potential therapeutic approach to ameliorate age-related diseases and extend healthy lifespan in humans.

NAD+ is essential for maintaining cellular energy balance and redox state. NAD+ is continuously converted by three types of NAD+ -consuming enzymes: NAD+ hydrolases, also known as the NAD+ enzymes (including CD38, CD157 (also known as bone marrow stromal cell antigen1, BST-1), and sterile alpha and TIR motif containing 1 (SARM1)), sirtuins (Revollo et al., 2004; Yang et al., 2007; Bowlby et al., 2012; Bruzzone et al., 2009; Van Gool et al., 2009; Koltai et al., 2010) and the poly (ADP-ribose) polymerases (PARPs) (Pillai et al., 2005). Metabolites of the NAD pathway play an important roles in signaling, post-translational modifications, epigenetic changes, and the regulation of RNA stability (Rodgers et al., 2008; Van der Horst et al., 2004; Bordone et al., 2006; Luo and Kraus, 2012). These enzymes utilize NAD+ as a substrate or cofactor and niacinamide (NAM) as a by-product. To maintain NAD+ levels, NAM can recycled NAD+ via the NAM salvage pathway. Additionally, some cells, mainly in the liver, can synthesize NAD+ dietary sources from peptides *de novo*. As a result, NAD+ is continuously synthesized, catabolized and circulated in the cell to maintain the stability of intracellular NAD+ levels (Figure 3).

**FIGURE 4:**
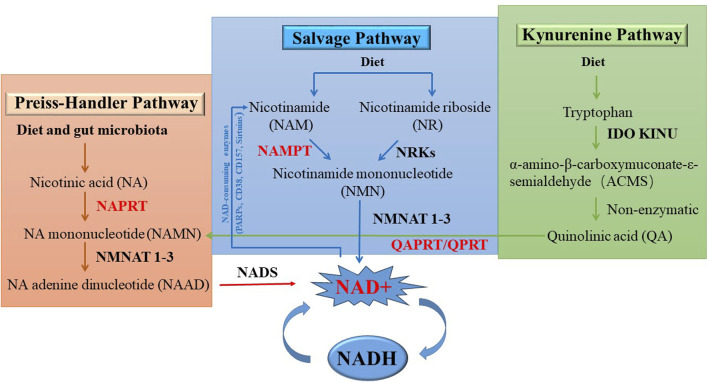
NAD+ biosynthetic pathways.

As shown in Figure 4, NAD+ can be synthesized from NAM, tryptophan or nicotinic acid (NA) through three distinct NAD biosynthesis pathways: the *de novo* pathway (also known as the Kynerunine pathway), the Preiss-Handler pathway, and the Salvage pathway (Chiarugi et al., 2012; Verdin, 2015). Different tissues follow the given pathways based on the availability of precursors (Canto et al., 2015; Shats et al., 2020; Liu et al., 2018). The *de novo* pathway initiates with tryptophan and goes through a series of enzymatic reactions to produce quinolinic acid (QA), which is converted into nicotinic acid mononucleotide (NAMN) by quinolinic phosphate ribosyl transferase (QAPRT/QPRT) (Bogan and Brenner, 2008). In the Preiss-Handler pathway, the phosphoribosyl group is transferred to nicotinic acid (NA) by nicotinic acid phosphoribosyltransferase (NAPRT), resulting in the production of NAMN. Therefore, NA is considered to be a precursor unit for NAD synthesis in the Preiss-Handler pathway (Preiss and Handler, 1958a; Preiss and Handler, 1958b). In the final step of the Preiss-Handler pathway, NAD synthetase (NADSYN) uses glutamine as a nitrogen donor to catalyze nicotinic acid adenine dinucleotide (NAAD) to NAD. In the Salvage pathway, NAD is produced from NAM, which is the final product of NAD-consuming enzymes. NAMPT is the rate-limiting enzyme in this pathway, catalyzing the conversion of NAM to NMN. In addition, mononucleotides NMN and NAMN can be produced by the phosphorylation of nicotinamide nucleoside (NR) and nicotinate nucleoside (NAR) by nicotinamide riboside kinase (NMRK1/2) (Bieganowski and Brenner, 2004; Tempel et al., 2007). NAMN and NMN are converted to the corresponding nicotinic acid adenine dinucleotide (NAAD) and NAD by the nicotinic acid mononucleotide adensine transferase (NMNAT 1-3) (Berger et al., 2005; Lau et al., 2009). The Salvage and Preiss-Handler pathways share the NMNAT 1-3 enzymes, which catalyze the final critical step in NAD synthesis. There are three subtypes of NMNAT: NMNAT 1, which is found in the nucleus; NMNAT two exists in the cytoplasm and Golgi apparatus; and NMNAT three is expressed in the mitochondria and cytoplasm (Figure 4).

NAM is derived from the diet (Bogan and Brenner, 2008; Trammell et al., 2016), and can be produced through the activity of various NAD hydrolases (including sirtuins, PARPs and CD38, etc.), which were tightly coupled with the Salvage pathway and play a role in the inflammation, cell growth, and bioenergetics (Magni et al., 2004), degrading NAD and producing the byproducts of NAM (Sauve, 2007; Quarona et al., 2013). Sirtuins have received widespread attention for their regulation of key metabolic pathways, stress responses, and the biology of aging. The sirtuin family comprises seven genes and proteins with unique subcellular localizations, enzymatic activities, and downstream targets, which affecting organel-specific functions and cellular metabolism. The human PARP family consists of 17 members. Of all PARPs, only PARP1, PARP2, and PARP3 are localized in the nucleus, where they respond to early DNA damage and play a key role in the DNA repair. CD38 and CD157 are multifunctional ectonucleotide enzymes with both glycohydrolase and ADP-ribosyl cyclase activities (Houtkooper et al., 2010; Chiarugi et al., 2012; Verdin, 2015).

## 5 SIRTs and NAD+

Sirtuins (SIRTs) are a family of nicotinamide adenine dinucleotide (NAD) -dependent histone deacetylases (HDACs), a group of evolutionally-conserved enzymes involved in post-translational modifications of proteins, including deacetylation, polyADP ribosylation, depropionylation, and lipoamidination. SIRTs are found in many cells and various organisms, and they has been discovered and explored in mammals over the past 2 decades (Chen B. et al., 2015). So far, seven members of this family have been identified in mammals: SIRT1-7, each member contains a conserved NAD-binding and catalytic domain (their N-terminus and C-terminus are distinct), known as the sirtuin core domain, which leads to distinct catalytic functions, subcellular localizations and substrate specificities. Meanwhile, sirtuin family members are capable of sensing cellular NAD+ levels (Bonkowski and Sinclair, 2016).

**FIGURE 5:**
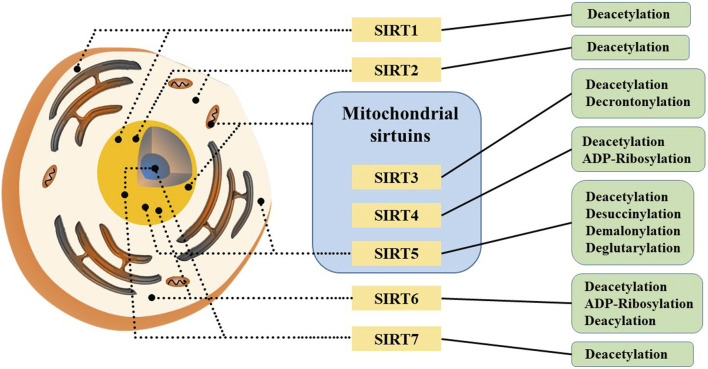
Subcellular localization and catalytic capacity of mammalian sirtuins.

SIRT1 is the founding member of the mammalian sirtuin family, primarily found in the cell nucleus and a small fraction present in the cytoplasm (Figure 5). It has been shown to play crucial roles in the process of development, cellular aging and cell death processes (Bai and Zhang, 2016; Wilking et al., 2014; Yu and Auwerx, 2010). Notably, NAMPT activates SIRT1 by increasing NAD+ levels and decreasing NAM levels (Menssen et al., 2012). SIRT1 exerts anti-aging effects and functions as a deacetylase that inhibits HIF-1α, a factor essential for activating the Warburg effect (Liberti and Locasale, 2016). Beyond hypoxia inducible factor-1α (HIF-1α), SIRT1 regulates other factors such as protein 53 (p53), myelocytomatosis viral oncogene homolog (c-Myc), forkhead box O3 (FOXO3), BCL2-associated X protein (BAX) and nuclear factor kappa-B (NF-κB). p53 plays a critical role in tumor suppression by inducing cell cycle and apoptosis (Behrouzfar et al., 2017). c-Myc is an oncogene that regulates genes involved in metabolic pathways like glycolysis, lactate production, glutamine metabolism and fatty acid synthesis, and regulates SIRT1 activity by inducing NAMPT expression and inhibiting deleted in breast cancer 1 (DBC1). SIRT1 regulates deacetylation activation of c-Myc through positive feedback (Menssen et al., 2012). In addition, SIRT1 deacetylates and activates FOXO3, which participates in oxidative stress resistance by upregating antioxidant proteins (Kennedy et al., 2016; Zhao et al., 2014). These data confirm that the carcinogenic effects of SIRT1 are largely depends on NAD+ and NAMPT activity.

SIRT2 is mainly located in the cytoplasm but also found in the nucleus, where it deacetylates H4K16, and involved in regulating the cell cycle. As a major consumer of intracellular NAD+, SIRT2 inhibits the peroxidase activity of Peroxiredoxin-1 (Prdx-1) through deacetylation, sensitizing breast tumor cells to increased reactive oxygen species (ROS) levels (Fiskus et al., 2016). Another target of SIRT2 is HIF-1α in the cytoplasm, promoting hydroxylation and degradation of HIF-1α, and inhibiting hypoxia-induced tumor growth (Seo et al., 2015). Studies indicate that SIRT2’s targets play roles in ROS-mediated pathways, including metabolic enzymes like glucose-6-phosphate dehydrogenase (G6PD), phosphoglycerate mutase 2 (PGAM2) and NF-κB (Gomes et al., 2015). Under oxidative stress conditions, SIRT2 has been shown to deacetylate and activate G6PD, a critical enzyme in the pentose phosphate pathway that produces nicotinamide adenine dinucleotide phosphate (NADPH) in the cytoplasm (Wang et al., 2014). Similarly, oxidative stress conditions cause PGAM2 to be deacetylated and activated by SIRT2, facilitating cellular responses to stress (Xu et al., 2014). Furthermore, SIRT2 activates NF-κB, which plays a pivotal role in regulating ROS in cells (Pais et al., 2013). NF-κB plays a dual role in regulating ROS by targeting enzymes that promote ROS production, such as NADPH oxidase, xanthine oxidoreductase, induced-nitric oxide synthase, cyclooxygenase-2, and cytochrome p450 enzymes. In summary, SIRT2 plays a crucial role in regulating oxidative stress responses, influencing various metabolic and signaling pathways through its deacetylase activity and interaction with key cellular regulators. Under conditions of excess nutrition, SIRT2 activity is decreased, increasing PKM2 acetylation and enzymatic activity. It is conducive to the production of lactic acid, while reducing the accumulation of pyruvate, forming a metabolic state similar to the Warburg effect. Conversely, in the absence of adequate nutrition, SIRT2 and other sirtuins are activated, leading to deacetylation of multiple downstream targets, including PKM2. This activates PKM2 and facilitates the accumulation of pyruvate, which provides nutrients for substrates used in the Krebs cycle and oxidative phosphorylation. Therefore, SIRT2 plays a key role in glucose metabolism (Park et al., 2016).

SIRT3 is mainly located in mitochondria, but also located in the nucleus, translocating to mitochondria during DNA damage to facilitate derepression of mitochondria-related genes (Ozden et al., 2014). SIRT3 inhibits cell apoptosis, promotes cell growth, increases glycolytic metabolism, promotes mitochondrial DNA repair, and increases cell resistance to environmental stress (Yang et al., 2020; Yang et al., 2016; Torrens-Mas et al., 2017a; Torrens-Mas et al., 2017b). Recent studies have shown that SIRT3 regulates mitochondrial metabolism and its collaborative effect with SIRT1 in extending lifespan in experimental animals. Notably, SIRT3 is the only member of the sirtuin family with direct evidence suggesting it can extend human lifespan. It has been found that loss of SIRT3 increases the production of ROS and stabilizes the expression of the transcription factor HIF-1α. SIRT3 also influences ROS production by modulating enzymes involved in mitochondrial oxidative phosphorylation (OXPHOS) pathway, thereby directly impacting cellular health (Haigis et al., 2012). Furthermore, SIRT3 plays a crucial role in repairing mitochondrial DNA and protecting mitochondrial integrity. It also regulates mitochondrial function through NAD+ levels, which can help protect liver and kidney from diseases and injuries (Morigi et al., 2015).

SIRT4 is a mitochondrial sirtuin, functioning as an NAD+ -dependent ADP-ribosyltransferase, highly expressed in the heart, kidney, liver and brain (Anderson et al., 2017). In glutamine catabolism, SIRT4 is the first target of glutamate dehydrogenase (GDH), which controls amino acid-stimulated insulin secretion by regulating the oxidative metabolism of glutamine and glutamate (Haigis et al., 2006). Apart from glutamine metabolism, SIRT4 inhibits β-oxidation of fatty acids, unlike SIRT3 and SIRT5. Through deacetylation of malonyl-coA decarboxylase (MCD), SIRT4 suppresses and catalyzes the conversion of malonyl-CoA to acetyl-CoA, essential for fatty acid oxidation.

SIRT5 is primarily located in mitochondria, with a small fraction found in the cytoplasm and nucleus. Unlike SIRT3 and SIRT4, SIRT5 exhibits weaker deacetylase activity (Du et al., 2011). Recent studies indicate that SIRT5 catalyzes desuccinylation, deglutarylation, and demalonylation of mitochondrial enzymes that are involved in various metabolic pathways such as glycolysis, fatty acid oxidation and the urea cycle. SIRT5 is highly expressed in tissues like the brain, heart, liver and lymphocytes. Lin et al. demonstrated that SIRT5 binds to superoxide dismutase 1 (SOD1) and desuccinylates SOD1, increasing SOD1 activity. Studies have found that cells transfected with SIRT5 have reduced ROS levels, indicating that SIRT5 inhibits oxidative stress in cells (Liang et al., 2017). Quantitative proteomic analysis has identified SIRT5’s interaction with enzymes primarily associated with glycolysis and gluconeogenesis, particularly glyceraldehyde-3-phosphate dehydrogenase (GAPDH), through desuccinylation. Experimental evidence indicates that loss of SIRT5 reduces glycolytic flux (Nishida et al., 2015). However, SIRT5 has also been reported to inhibit glycolysis by desuccinic pyruvate kinase M2 (PKM2), an enzyme involved in the final step of glycolysis, which protects tumor cells from oxidative stress (Wang et al., 2017; Ye et al., 2017). Furthermore, the function of SIRT5 in metabolic control is dependent on the environment, as it can either promote or inhibit specific metabolic processes based on cell type and nutrient availability. In summary, SIRT5’s diverse enzymatic activities contribute significantly to metabolic regulation, impacting cellular metabolism under both physiological and stress conditions.

SIRT6 primarily resides in the nucleus and exerts its effects through NAD+ -dependent deacetylation of H3K9 and H3K56. By inhibiting PKM2, SIRT6 suppresses the Warburg effect, thereby regulating glucose metabolism and homeostasis (Bhardwaj and Das, 2016). Although SIRT6 was originally described as a unique ADP-ribosyltransferase (Liszt et al., 2005), recent findings that histones, DNA repair enzymes and DNA polymerase β (polβ) were deacetylated *in vitro*, influencing the efficiency of DNA repair (Mostoslavsky et al., 2006). These functions highlight SIRT6’s critical roles in cell metabolism, gene expression regulation and DNA repair.

SIRT7 is primarily located in the nucleus, specifically in the nucleolus, where it interacts with RNA Pol I and histones, actively regulating the transcription of ribosomal DNA (rDNA), which constitutes approximately 60% of total transcription in metabolically active mammalian cells (Grabowska et al., 2017). It has been found that SIRT7 mRNA expression is different in all tissues, and higher expression in tissues with higher metabolic activity. Overexpression of SIRT7 enhances RNA Polymerase I (RNA Pol I) mediated transcription in a NAD+ -dependent manner, while knockdown or inhibition of SIRT7 decreases this transcriptional (Ford et al., 2006). This suggests that SIRT7 may regulate rDNA transcription by sensing cellular NAD+ levels, linking cellular energy status to rRNA synthesis and ribosome production. Research has found that SIRT7 regulates mitochondrial homeostasis through deacetylation of GA binding protein transcription factor beta subunit 1 (GABPβ1), a subunit of the complex that regulates several key mitochondrial genes (Ryu et al., 2014). Furthermore, the absence of SIRT7 inhibits cell proliferation and induces apoptosis, indicating its potential role in aging and/or age-related diseases.

## 6 The role of NAMPT in disease

### 6.1 The role of NAMPT in inflammatory diseases

NAMPT has been identified as a universal biomarker for chronic inflammation. Chronic inflammatory diseases such as rheumatoid arthritis (RA), lung injury, inflammatory bowel disease (IBD), psoriasis and atopic dermatitis (AD). NAMPT acts as a growth factor and stimulates the proliferation of pre-B-cells (Samal et al., 1994). Jia et al. (2004), it was demonstrated for the first time that NAMPT plays a role as a cytokine, whose expression is upregulated in a variety of acute and chronic inflammatory diseases. Studies have shown that IL-1β induces the expression of NAMPT in human neutrophils and NAMPT can prevent the apoptosis of neutrophils under the inflammatory stimulation. However, inhibition of NAMPT enzyme activity impimped NLRP3-dependent and independent inflammatory responses (TNF-α and IL-6), and protein phosphorylation downstream of the TLR4 signaling pathway (Yang et al., 2019).

It has been found that NAMPT induce the expression of IL-6, matrix metalloproteinase 1 (MMP-1) and MMP-3 in synovial fibroblasts of rheumatoid arthritis (Nowell et al., 2006). Experiments in mouse models showed that IL-6 deficiency impairs inflammatory infiltration and NAMPT expression. In acute lung injury, NAMPT is upregulated at mRNA and protein levels (Ye et al., 2005; Camp et al., 2015). In animal models of ischemia/reperfusion-induced lung injury, NAMPT has shown protective anti-inflammatory effects (Wu et al., 2017). At the same time, high levels of NAMPT have been found in the serum, colonic tissue, and leukocytes of IBD (Moschen et al., 2007; Neubauer et al., 2019). The main source of NAMPT in the colon and visceral adipose tissue of IBD patients is located in macrophages in submucosal adipocytes, dendritic cells, and epithelial cells (Moschen et al., 2007). Inhibition of NAMPT expression reduced cytokine production in IBD-derived immune cells (Gerner et al., 2018).

Psoriasis is a non-infectious chronic inflammatory skin disease with a global prevalence of 0.1%–3%, characterized by recurrent episodes that seriously affect the mental and physical health of patients. The cytokines and chemokines produced in the lesions reach the blood, so the patient may suffer from comorbidities, especially IL-1β and TNF-α causing cardiovascular complications, metabolic syndromes (such as obesity, dyslipidemia, atherosclerosis, and type 2 diabetes), and autoimmune diseases. It has been reported in the literature that NAMPT is overexpressed in peripheral blood mononuclear cell (PBMC) of psoriasis patients, but it has returned to normal during the cure period (Koczan et al., 2005). Comparing gene expression in skin samples of normal and psoriasis patients (lesions and non-lesions), NAMPT overexpression appeared in lesions skin (Xie et al., 2014). A meta-analysis found that the levels of eNAMPT was no significant difference in the serum of psoriasis patients and controls (Bai et al., 2018), but its levels positively correlated with psoriasis area and severity index (PASI) scores (Chyl-Surdacka et al., 2020) and duration (Ismail and Mohamed, 2012). In contrast, patients with psoriatic arthritis had high serum levels of NAMPT, but their levels were not associated with disease activity (Dikbas et al., 2016). Keratinocytes, neutrophils, dendritic cells, and T cells play a critical role in the dermal and epidermal pathology of psoriasis (Boehncke and Schön, 2015). eNAMPT has been shown to mediate the production of cathelicidin antimicrobial peptides (CAMP), β-defensin-2, β-defensin-3, and S100A7 in human keratinocytes and imiquimote-induced mouse models of psoriasis (Hau et al., 2013). eNAMPT has also been shown to stimulate angiogenesis, migration, proliferation, invasion, and capillary tube formation in human umbilical vein endothelial cells (HUVECs) and human microvascular endothelial cells (HMECs) *in vitro*, as well as in rat and mouse angiogenic models (Boehncke and Schön, 2015; Bae et al., 2009; Kim et al., 2007; Kim et al., 2012; Lovren et al., 2009). Serum levels of eNAMPT are elevated in patients with AD.

### 6.2 The role of NAMPT in cardiovascular disease

Dahl et al. identified the relationship between NAMPT and cardiovascular disease, and found that the expression of NAMPT was enhanced in lipid macrophages in atherosclerotic lesions in patients with myocardial infarction (Dahl et al., 2007). Experiments in animal models exposed to a high-fat diet (HFD) suggested that NAMPT overexpression leads to worsening of atherosclerotic lesions and inflammation (Kong et al., 2019). Interestingly, NAMPT heterozygous knockdown prevented cardiac hypertrophy, but genetically modified mice with heart-specific NAMPT overexpression spontaneously developed cardiac hypertrophy (Byun et al., 2019; Pillai et al., 2013). It has been reported that eNAMPT triggers the Toll-like receptor 4/NOD-like receptor thermal protein domain associated protein three/interleukin 1β (TLR4/NLRP3/IL-1β) axis in the literature (Romacho et al., 2020), both NAMPT and SIRT1 protect the heart from ischemia/reperfusion. The analysis found that serum concentrations of NAMPT were much higher in patients with cardiovascular disease than in healthy individuals (Yu et al., 2019). High eNAMPT serum levels were also found in peripheral blood of patients with acute coronary syndrome.

### 6.3 The role of NAMPT in metabolic diseases

Studies have demonstrated a high expression of NAMPT in visceral adipose tissue, classifying as an adipokine (Fukuhara et al., 2005). Consequently, the role of NAMPT in obesity and related diseases has draw people’s attention. Various adipocyte models, including preadipocyte lines such as 3T3-L1 and SGBS, along with human primary adipocytes, have been shown to secrete NAMPT into the supernatant via non-classical pathway (Tanaka et al., 2007). This identifies adipose tissue as one of the primary sources of extracellular NAMPT (eNAMPT). The study found increased expression levels of several metabolic factors in obese individuals, which have been shown to affect the expression levels of NAMPT. During adipogenesis, the expression level of NAMPT mRNA is increased and stimulated by insulin resistence-inducing factors such as IL-6, and TNF-α. The expression of NAMPT in adipocytes was also upregulated under hypoxic conditions (Garten et al., 2015; Kralisch et al., 2005; Kim et al., 2014). The pro-inflammatory effects of eNAMPT on different cell types have been reported in the literature, including induction of nitric oxide synthase (Romacho et al., 2009), activation of extracellular signal-regulated protein kinase 1/2 (ERK1/2) (Kim et al., 2007), nuclear factor NF-κB (Romacho et al., 2009; Moschen et al., 2010), and cytokines such as TNF-α, IL-6, IL-1β (Moschen et al., 2010; Hector et al., 2007), trans forms growth factor β (Song et al., 2008), and monocyte chemotactic protein 1 (Sommer et al., 2010). In addition, eNAMPT increased the expression of peroxide-activating receptors in lipoprotein lipase and preadipocytes and fatty acid synthetase in differentially differentiated adipocytes, suggesting that eNAMPT is a regulator of lipid metabolism (Yang et al., 2010). The TNF-α stimulated mouse adipocytes and human hepatocytes with high levels of pro-inflammatory cytokine production showed insulin resistance induced by eNAMPT (Gouranton et al., 2014; Heo et al., 2019). In addition, neutrophils are thought to be the main source of NAMPT release in the blood (Martínez-Morcillo et al., 2021; Friebe et al., 2011).

### 6.4 The role of NAMPT in neurodegenerative diseases

Typical neurodegenerative diseases include Alzheimer’s disease (AD), Parkinson’s disease (PD), Huntington’s disease (HD), and amyotrophic lateral sclerosis (ALS). Neurodegenerative diseases are mainly related to mitochondrial dysfunction, inflammation and oxidative stress. Among them, oxidative stress is considered to be an important pathogenic factor in inducing cell proliferation, mitochondrial dysfunction, self-renewal and hypodifferentiation, and downregulation of NAD and NAMPT levels in neurodegenerative diseases. NAD is an essential coenzyme involved in energy production and redoxic metabolism, which can be generated *de novo* from tryptophan or recovered from NAM through NAMPT-dependent salvage pathways, and is closely related to mitochondrial energy metabolism. NAMPT-mediated NAD salvage pathway is the main synthetic pathway of NAD, and defects in the biosynthesis of NAD lead to the decline of NAD. Therefore, NAMPT is essential for maintaining NAD balance in the body.

Alzheimer’s disease (AD) is the most common neurodegenerative disease. The prevalence of AD increases significantly with age, primarily affecting older adults. Studies have shown that cytokines involved in mitosis, such as NRF1, NRF2, and TFAM, are associated with neurodegenerative diseases such as AD. Therefore, promoting mitosis may be an effective treatment for AD. Recent studies have shown that overactivation of the immune proteasome (IP) can trigger neuroinflammation and neuronal death (Sonninen et al., 2020). Neuroinflammation and oxidative stress can induce neurodegeneration (Kwon and Koh, 2020). By inhibiting inflammatory responses and oxidative stress, iNAMPT is functionally involved in neurodegenerative diseases.

Parkinson’s disease (PD) is the second common neurodegenerative disease in the world, severely affecting the normal life of middle-aged and elderly patients (Jankovic and Tan, 2020). It is generally believed that oxidative stress, chronic inflammation, and mitochondrial dysfunction are the main causes of PD (Pajares et al., 2020). Research has found that mitochondrial dysfunction is a key driver of Parkinson’s disease. iNAMPT can maintain cell metabolism, which in turn affects mitochondrial function. Increasing NAD through nicotinamide riboside, a precursor of NAD, improves mitochondrial function of patients’ neurons. iNAMPT can synthesize NAD from NAD precursors using the NAD biosynthetase NR kinase 1 (NRK1) (Schöndorf et al., 2018). There is increasing evidence that NSCs undergo cellular senescence under various stress conditions (Zeng et al., 2021). iNAMPT is particularly important for self-renewal, differentiation, and proliferation of NSPCs. Therefore, targeting iNAMPT will become a new research direction for PD therapy.

Amyotrophic lateral sclerosis (ALS) is a hereditary neurodegenerative disease in which the main symptoms include muscle spasms and weakness, contraction and atrophy of muscle bundles (Hardiman et al., 2017). At present, the complex pathogenesis of ALS has not been fully elucidated. Mitochondrial dysfunction, oxidative stress, metabolic disorders and neuroinflammation have been identified as potential pathological factors (Hardiman et al., 2017). Human superoxide dismutase 1 (hSOD1) is isolated from primary astrocytes in mice and can induce motor neuron death (Harlan et al., 2016; Obrador et al., 2021). Elevated levels of astrocyte mitochondrial NAD in ALS patients enhance resistance to oxidative stress and reverse the toxicity of co-cultured motor neurons (Obrador et al., 2021). iNAMPT is a rate-limiting enzyme in the NAD salvage pathway, and its overexpression upregates the mitochondrial level of NAD in astrocytes. Therefore, iNAMPT may be a potential therapeutic target for preventing astroglia-mediated motor neuron death in ALS patients.

## 7 NAMPT inhibitors

Due to abnormal proliferation and higher energy demand, tumor cells are more dependent on NAD+ than normal cells. NAMPT is an enzyme that plays a key role in the NAD+ biosynthesis pathway, and its inhibitors have shown potential in cancer therapy. In recent years, an increasing number of NAMPT inhibitors have been reported. FK866, is the earliest discovered NAMPT inhibitor, which selectively inhibits NAMPT, resulting in a decrease in NAD+ levels, and then inhibiting the growth of tumor cells (Hasmann and Schemainda, 2003). CHS828 is an effective NAMPT inhibitor that has been used in clinical trials for cancer treatment, but further development was halted due to toxicity and poor effectiveness (Olesen et al., 2008). GMX1777, a prodrug of CHS828, was designed to address solubility and pharmacokinetic issues, showing potent inhibitory activity *in vivo* (Binderup et al., 2005). OT-82, a novel NAMPT inhibitor that is currently in clinical trials, inducing cell apoptosis through NAD and ATP depletion (Korotchkina et al., 2020). GNE617 is a NAMPT inhibitor that is structurally different from FK866 and acts by binding to the active site of NAMPT (Zheng et al., 2013b). KPT-9274, a dual inhibitor that simultaneously inhibits both NAMPT and PAK4, has shown strong effects against a variety of solid tumors and hematological malignancies in clinical trials (Abu Aboud et al., 2016). Several studies are developing dual-target inhibitors that can simultaneously inhibit NAMPT and other targets (such as HDAC), which may provide more effective therapeutic effect. STF-31 not only inhibits NAMPT, but also inhibits GLUT1, showing an inhibitory effect on tumor cells (Kraus et al., 2018). Antibody-drug conjugates (ADCs) are a strategy for directly delivering potent drugs to tumor tissue, potentially improving the therapeutic index of NAMPT inhibitors (Neumann et al., 2018). The development and research of these inhibitors provide new strategies and methods for cancer treatment. Unfortunately, only a few small molecule inhibitors of NAMPT have progressed to clinical studies, and the rest are still in the preclinical stage due to obvious adverse reactions or insufficient *in vivo* experimental data, indicating that further studies are needed to improve their efficacy and safety.

## 8 Conclusion

In recent years, the research on the biological function of NAMPT, particularly its extracellular roles, have made great progress. The NAMPT-NAD-SIRT cascade has been identified as a powerful intrinsic defense system against energy expenditure and neuronal death in neurodegenerative diseases. During various metabolic disorders and aging, the expression level of NAD are decreased. The salvage pathways, primarily dependent on the rate-limiting enzyme NAMPT, are crucial for maintaining human NAD. NAMPT supplies substrates for NAD-dependent enzymes involved in regulating cellular energy metabolism. NAMPT is released by different cell types in response to cellular stress and inflammatory signals, such as hypoxia, starvation, hyperglycemia and pro-inflammatory cytokines. Given that visceral fat is the primary tissue for NAMPT release, extracellular NAMPT may play a significant role in chronic inflammatory diseases and their complications, including obesity, metabolic syndrome, cardiovascular diseases and diabetes. Extracellular NAMPT not only acts as a systemic pro-inflammatory cytokine, but also increases the level of NAD+ expression when it reaches the inflammatory tissue, thus significantly enhancing the activity of PARPs and SIRT. Although studies in animal models suggest that NAMPT may be a promising therapeutic target for clinical intervention in chronic inflammatory diseases, its relevance needs to be further clarified.

## Supplementary material

SUPPLEMENTARY FIGURE S1
Crystal structure of visfatin.
