# Supplementary figures and images for: The function of nicotinamide phosphoribosyl transferase (NAMPT) and its role in diseases
Source: Front Mol Biosci. 2024 Oct 24;11:1480617. doi: 10.3389/fmolb.2024.1480617 (PMC11540786; doi:10.3389/fmolb.2024.1480617)

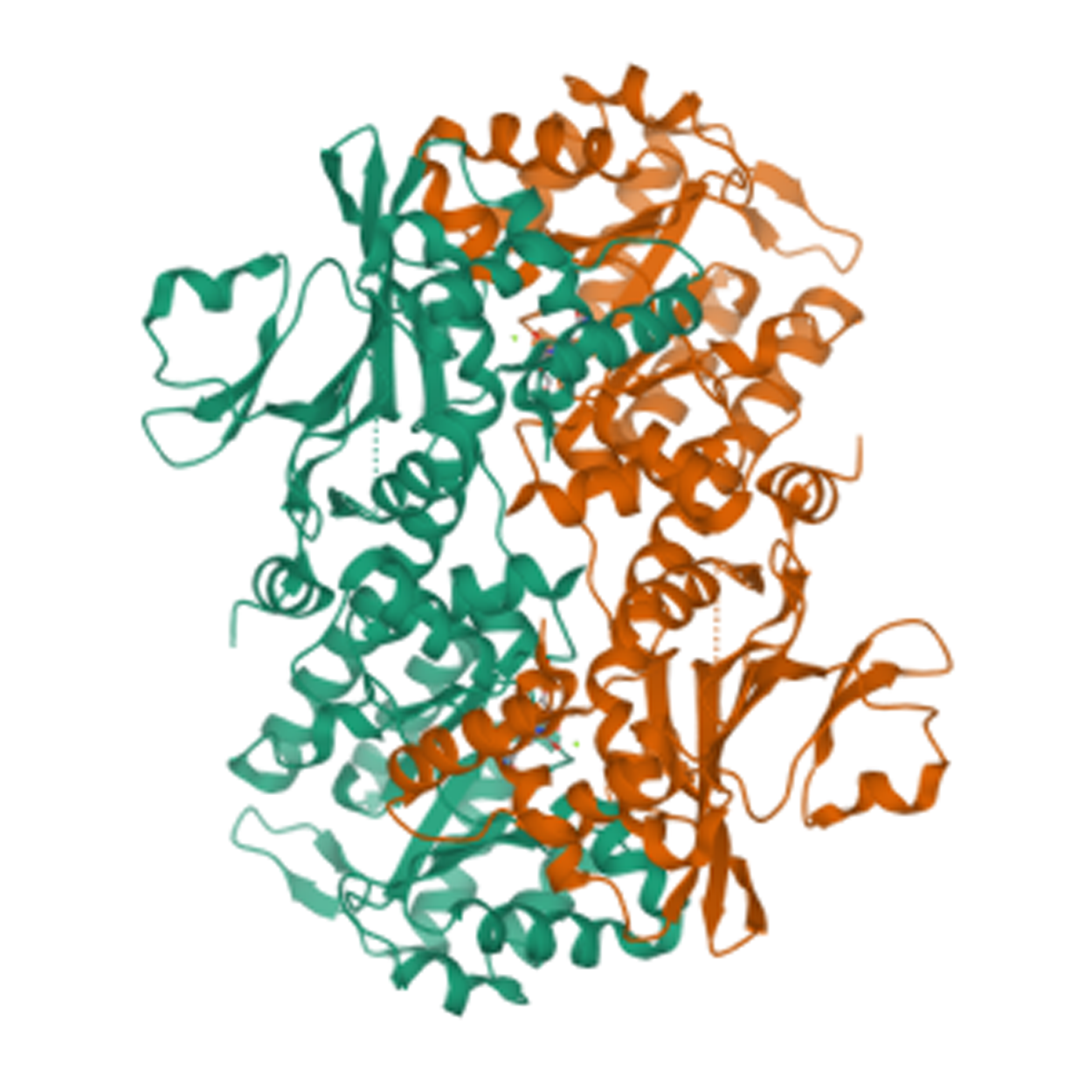

Supplement: Supplementary file 1 [file Image1.tif]
